# Supplementary material for: Teach your microscope how to print: low-cost and rapid-iteration microfabrication for biology
Source: Lab Chip. 2025 Jul 14;25(16):4091–105. doi: 10.1039/d5lc00181a (PMC12257287; doi:10.1039/d5lc00181a)
Supplement: LC-025-D5LC00181A-s001 [file LC-025-D5LC00181A-s001.zip › fig-s-1.pdf]

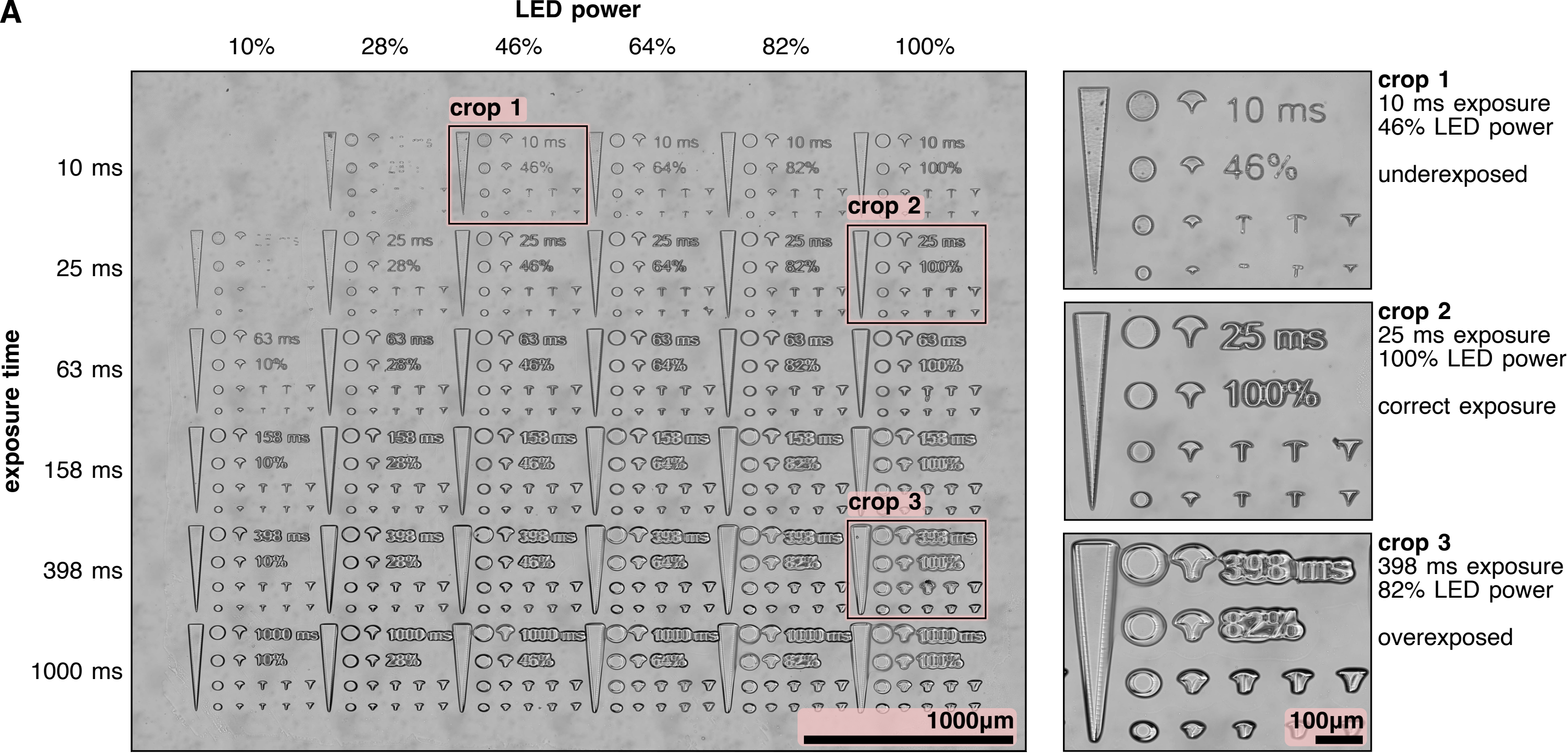

A
LED power
10%
28%
46%
64%
82%
100%
exposure time
10 ms
25 ms
63 ms
158 ms
398 ms
1000 ms
crop 1
crop 2
crop 3
1000µm
100µm
crop 1
10 ms exposure
46% LED power
underexposed
crop 2
25 ms exposure
100% LED power
correct exposure
crop 3
398 ms exposure
82% LED power
overexposed
