# Supplementary figures and images for: Teach your microscope how to print: low-cost and rapid-iteration microfabrication for biology
Source: Lab Chip. 2025 Jul 14;25(16):4091–105. doi: 10.1039/d5lc00181a (PMC12257287; doi:10.1039/d5lc00181a)

**A**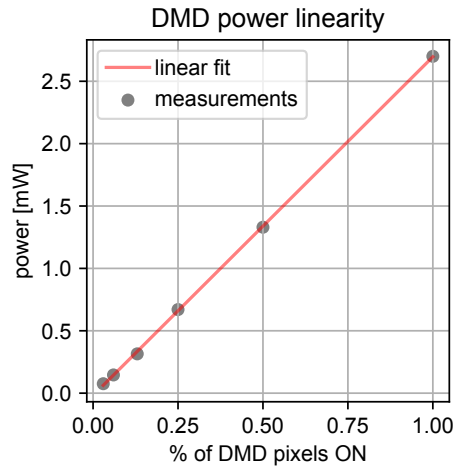

Supplement: LC-025-D5LC00181A-s001 [file LC-025-D5LC00181A-s001.zip › fig-s-10.pdf]

**A**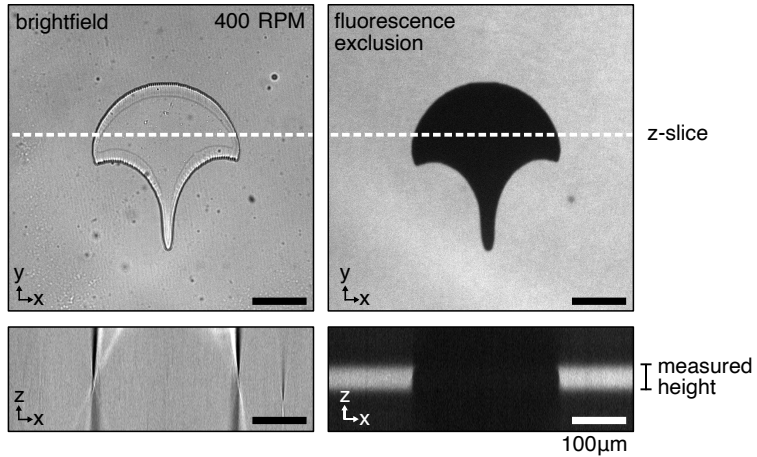**B**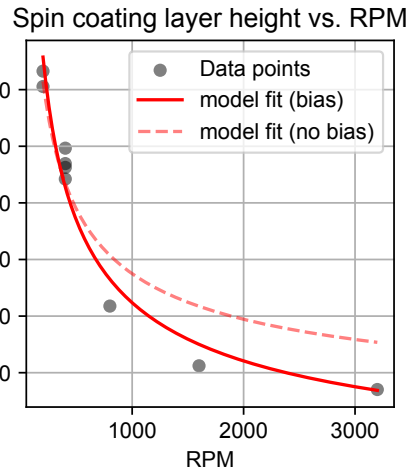

Supplement: LC-025-D5LC00181A-s001 [file LC-025-D5LC00181A-s001.zip › fig-s-2.pdf]

**A**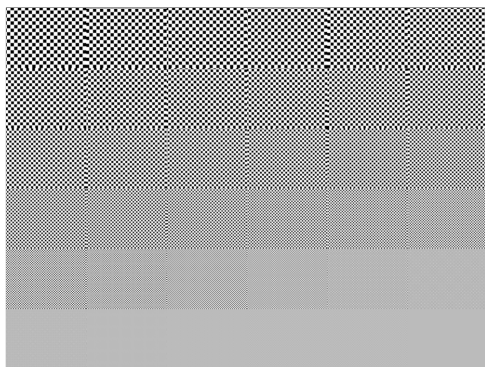**B**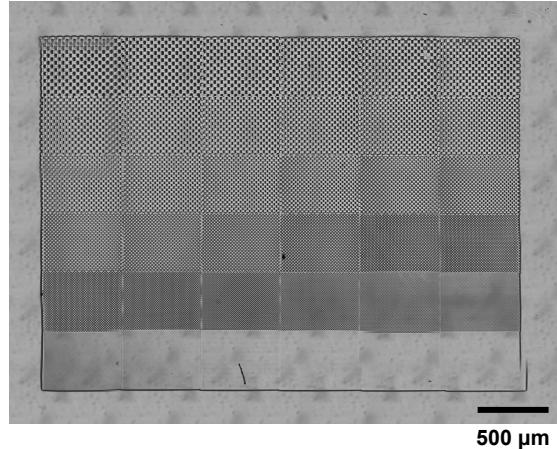500  $\mu\text{m}$ **C**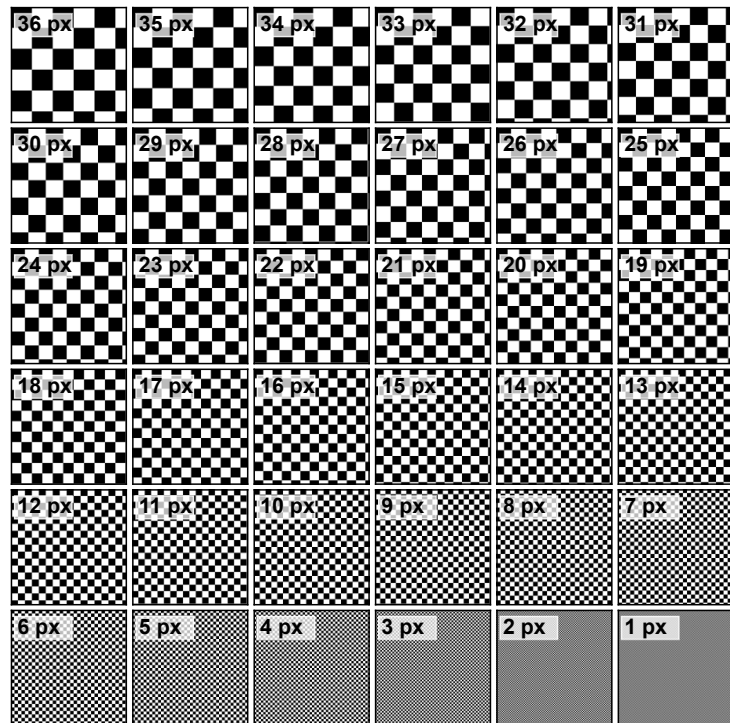**D**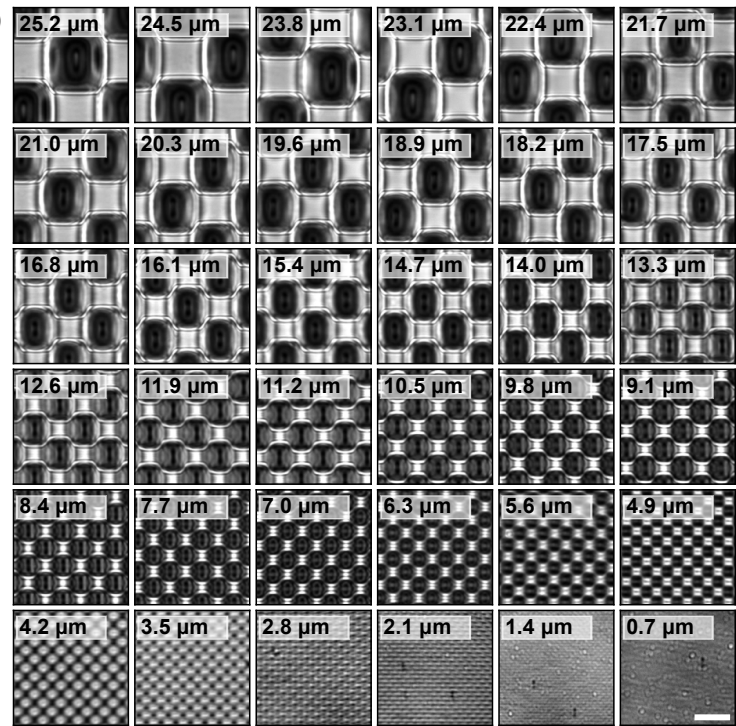10  $\mu\text{m}$

Supplement: LC-025-D5LC00181A-s001 [file LC-025-D5LC00181A-s001.zip › fig-s-3.pdf]

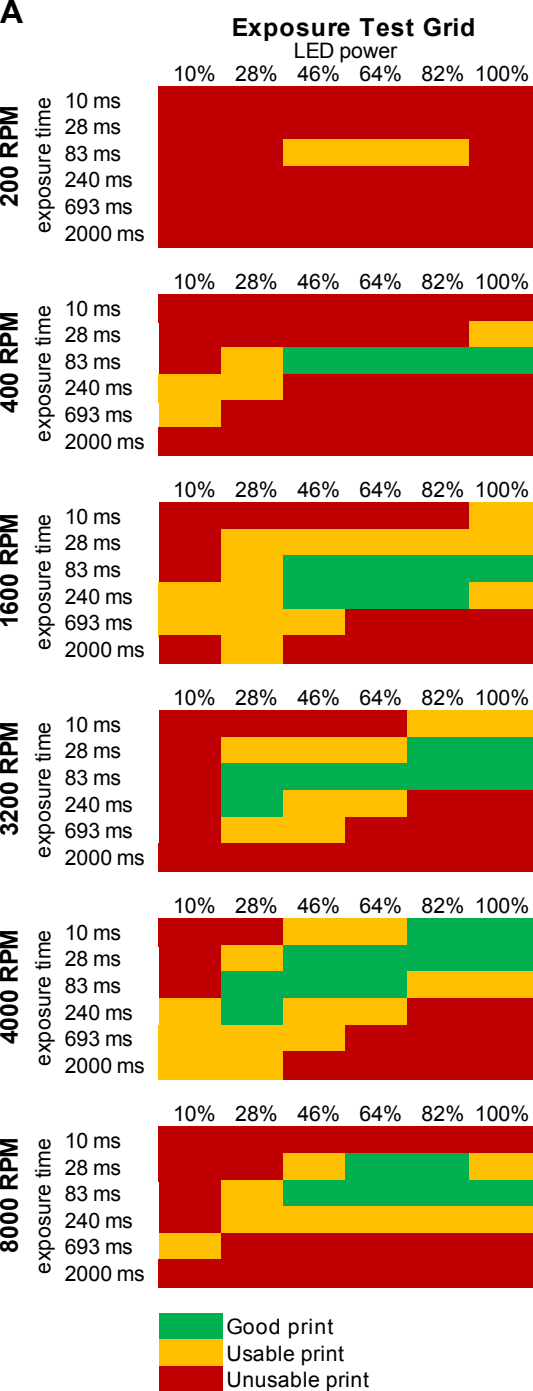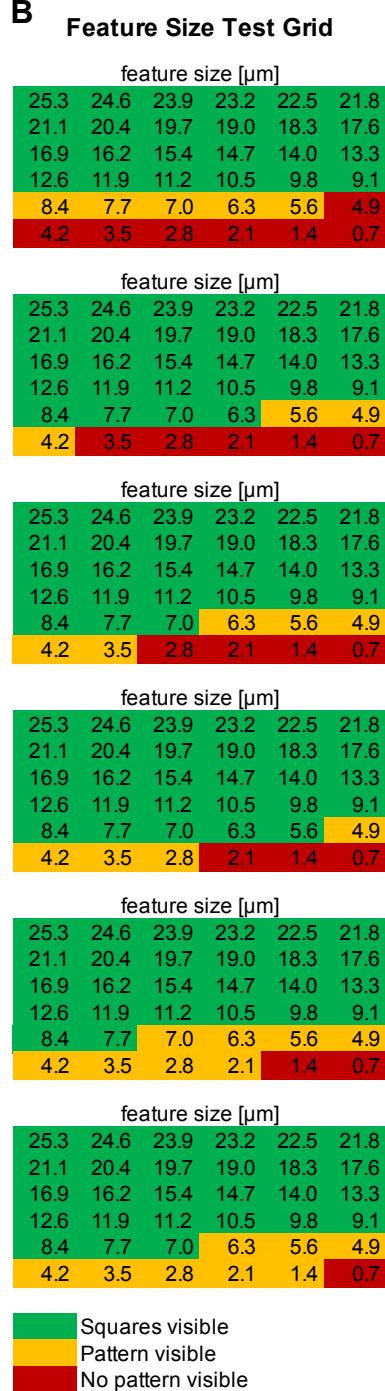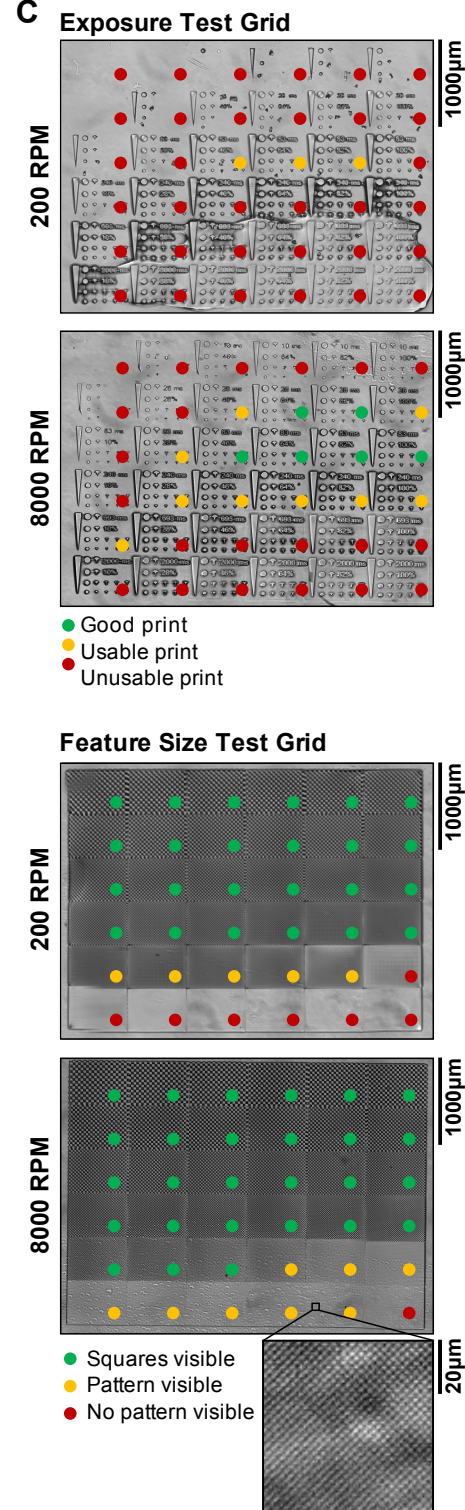

Supplement: LC-025-D5LC00181A-s001 [file LC-025-D5LC00181A-s001.zip › fig-s-4.pdf]

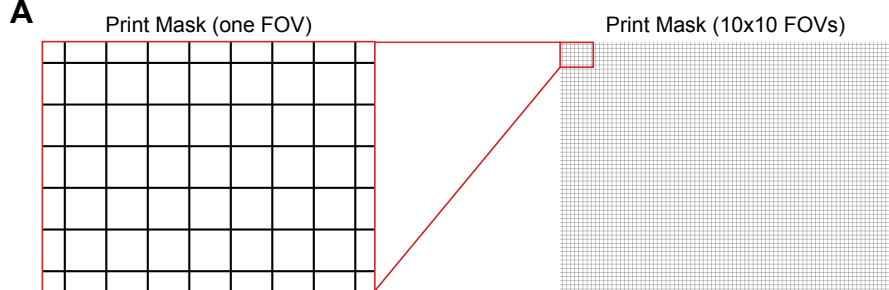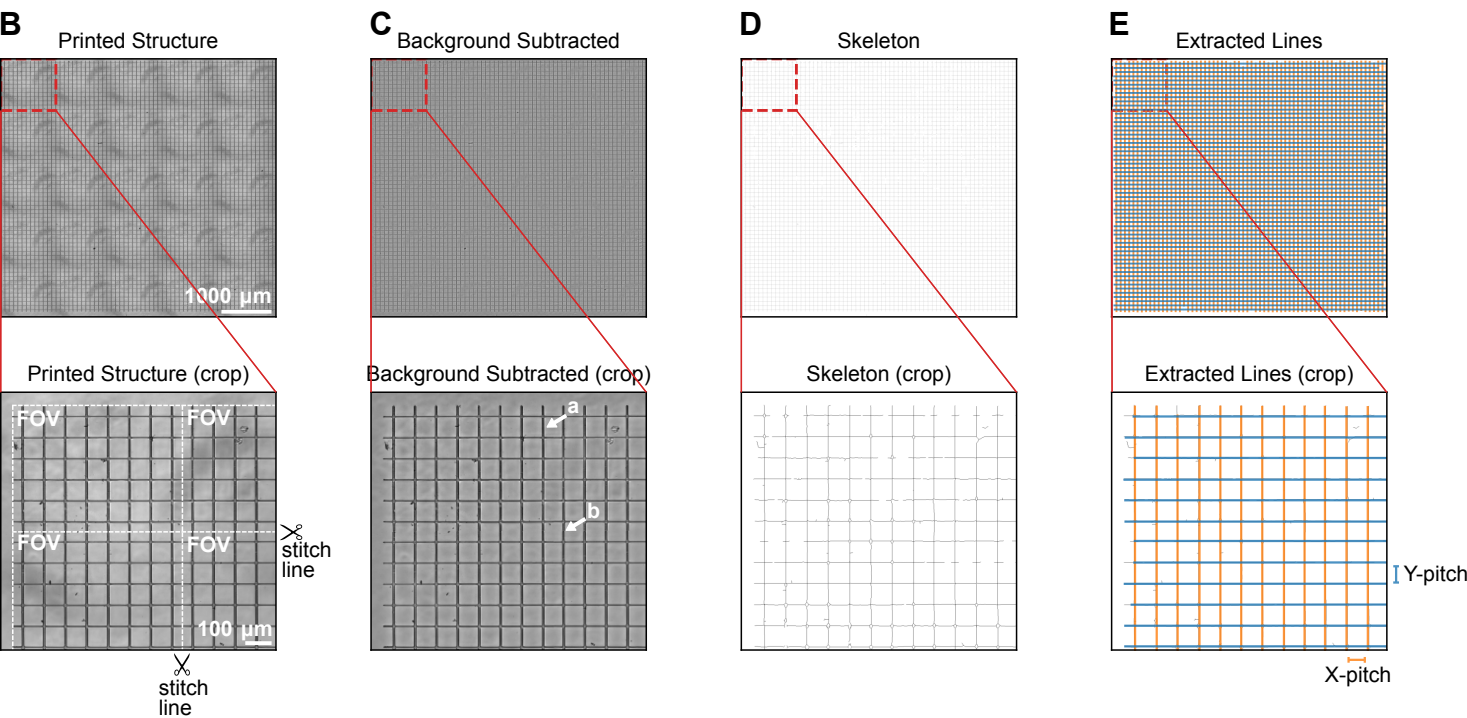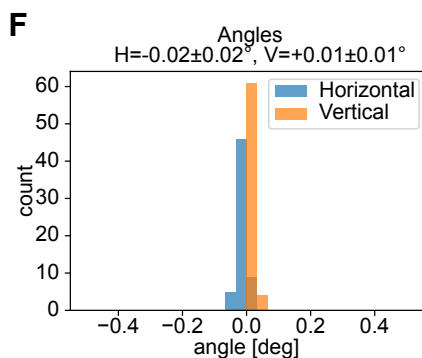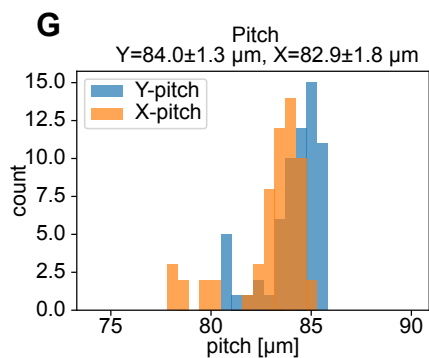

Supplement: LC-025-D5LC00181A-s001 [file LC-025-D5LC00181A-s001.zip › fig-s-5.pdf]

**A**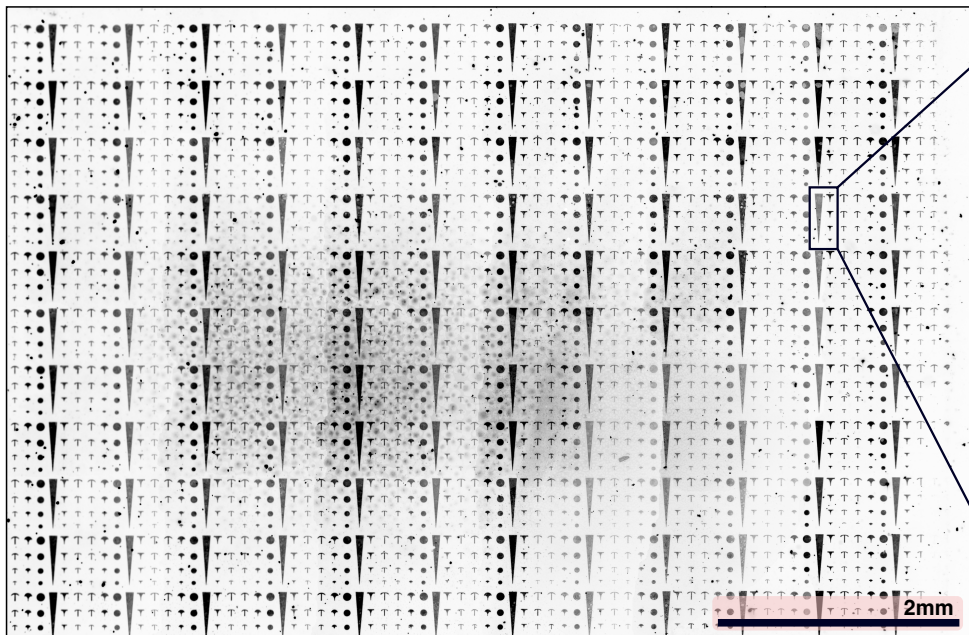**B**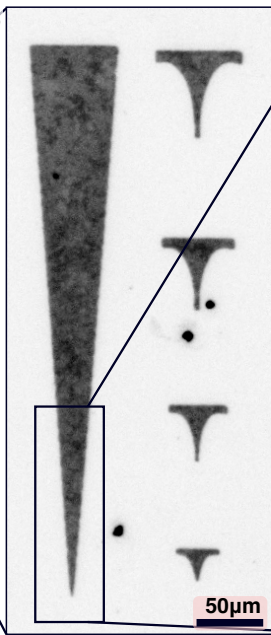**C**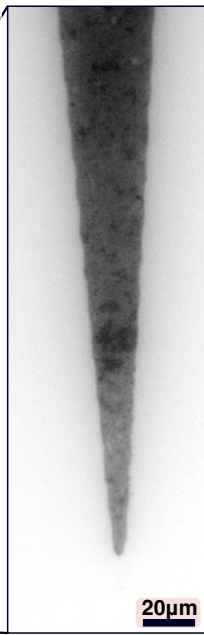

Supplement: LC-025-D5LC00181A-s001 [file LC-025-D5LC00181A-s001.zip › fig-s-6.pdf]

**A**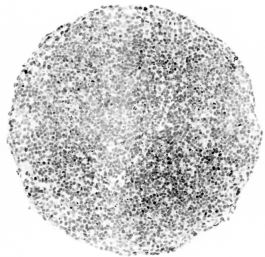**B**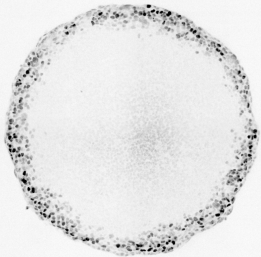**C**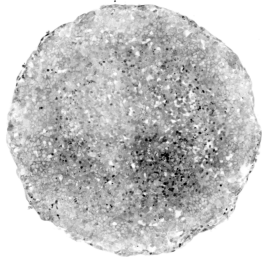**D**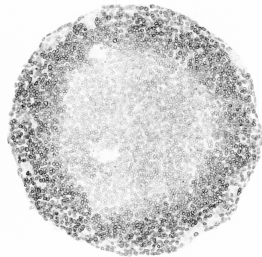

200  $\mu$ m

Supplement: LC-025-D5LC00181A-s001 [file LC-025-D5LC00181A-s001.zip › fig-s-7.pdf]

**A**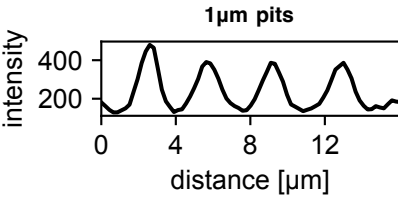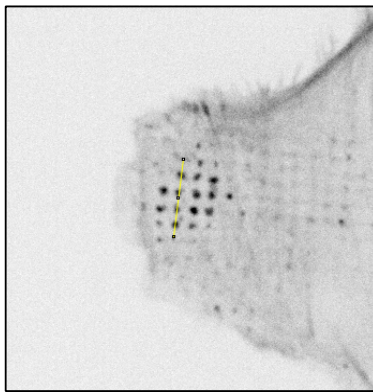**B**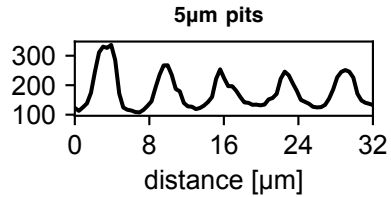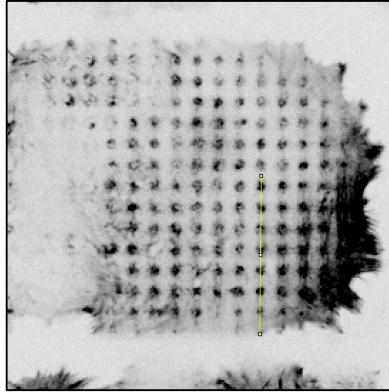

Supplement: LC-025-D5LC00181A-s001 [file LC-025-D5LC00181A-s001.zip › fig-s-8.pdf]

**A**

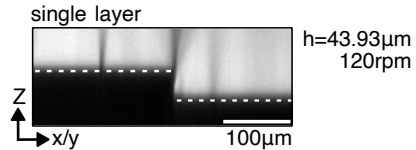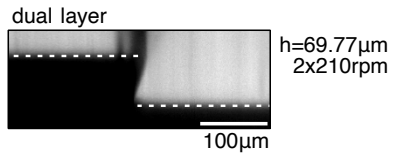

Supplement: LC-025-D5LC00181A-s001 [file LC-025-D5LC00181A-s001.zip › fig-s-9.pdf]
