# Supplementary material for: Teach your microscope how to print: low-cost and rapid-iteration microfabrication for biology
Source: Lab Chip. 2025 Jul 14;25(16):4091–105. doi: 10.1039/d5lc00181a (PMC12257287; doi:10.1039/d5lc00181a)
Supplement: LC-025-D5LC00181A-s002 [file LC-025-D5LC00181A-s002.pdf]

Supplementary Information (SI) for Lab on a Chip.
This journal is © The Royal Society of Chemistry 2025

Supplementary figures

S1 UV exposure calibration

S2 Z-layer height calibration

S3 X/Y resolution test using checkerboard pattern

S4 UV exposure calibration and printing accuracy for different layer heights

S5 FOV alignment accuracy over large areas

S6 Micropatterning of large areas

S7 Micropatterning of 2D gastruloid

S8 Actin clusters on microtopology

S9 2.5D printing

S10 Measuring DMD output power

Supplementary movies

M1 `M1_pits_migration_1um.mp4`
Timelapse movie of a cell migrating on microtopology with 1 µm sized pits (shown in Fig. 2A)

M2 `M2_pillars_migration_5um.mp4`
Timelapse movie of cells migrating on microtopology with 5 µm sized pillars (shown in Fig. 2C)

M3 `M3_constricted_migration.mp4`
Cell migrating trough a narrow constriction of a microfluidic chip (shown if Fig. 4F)

M4 `M4_C_elegans_microchamber.mp4`
Timeseries of a fluorescently tagged *C. elegans* worm confined in a microchamber. Complete lifecycle, from egg to adult (shown in Fig. 5F).

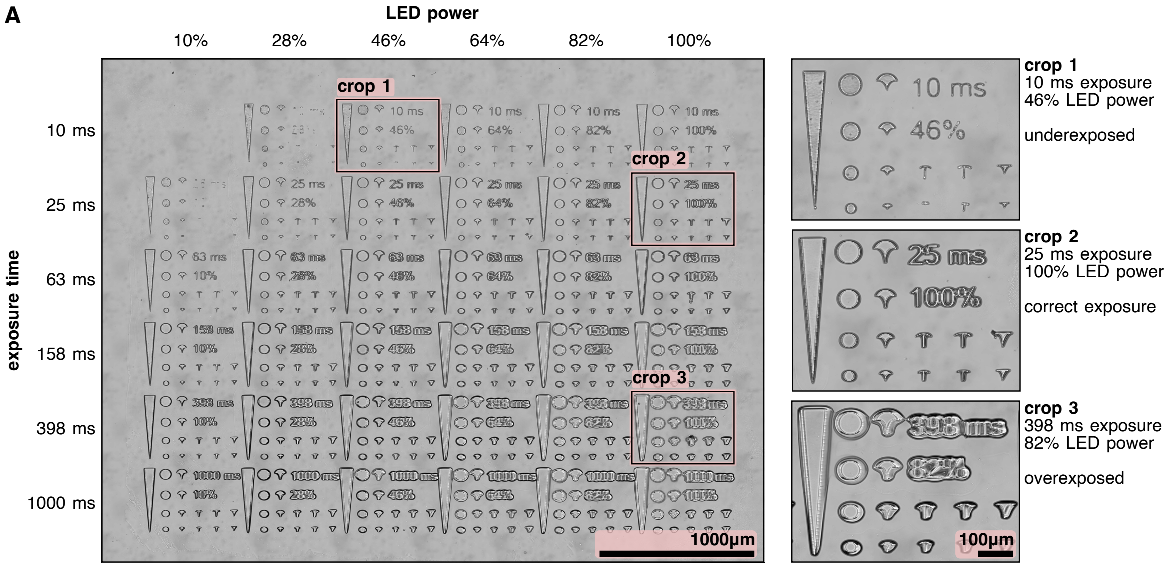

Fig. S1. UV exposure calibration: Empirical grid search for exposure time and led power parameters. Different settings can be tested automatically in a single step, simplifying the calibration procedure compared to classical approaches. This test shows linearly spaced LED power settings, from 10% to 100% and logarithmically spaced exposure times, from 10 ms to 1000 ms. Duration of the test (exposing, washing, inspection) was under 5 min. Three crops show underexposed, correct exposure and overexposed FOVs. Slide is spin coated at 3000 RPM.

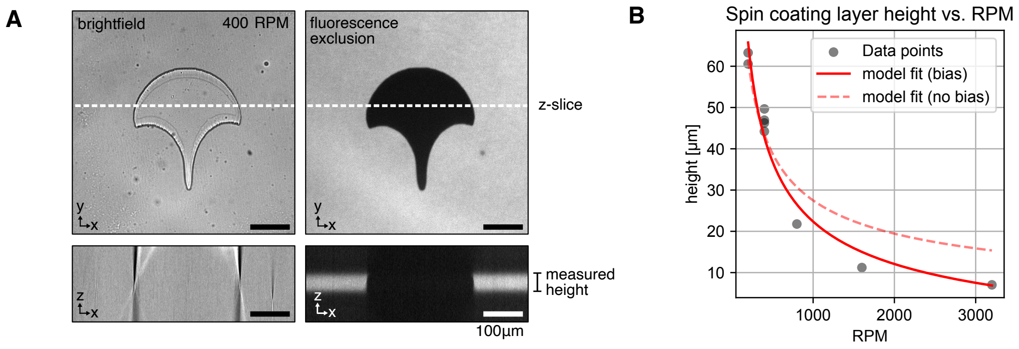

Fig. S2. Z-layer height calibration. A Sample FOV showing brightfield and fluorescence exclusion confocal z-stacks. Height is measured manually using an x/z projection in ImageJ. **B** Plot showing the layer heights measured for different RPM (200, 400, 800, 1600, 3200). Two models are shown, with constant bias: $h(RPM) = \frac{a}{\sqrt{RPM}} + h_0$, without bias: $h(RPM) = \frac{a}{\sqrt{RPM}}$. Model bias: $a = 1110.09,\ b = -12.74$ MAE: 3.60, RMSE: 4.07, R^2: 0.96. Model no bias: $a = 869.13$, MAE: 4.86, RMSE: 5.99, R^2: 0.90.

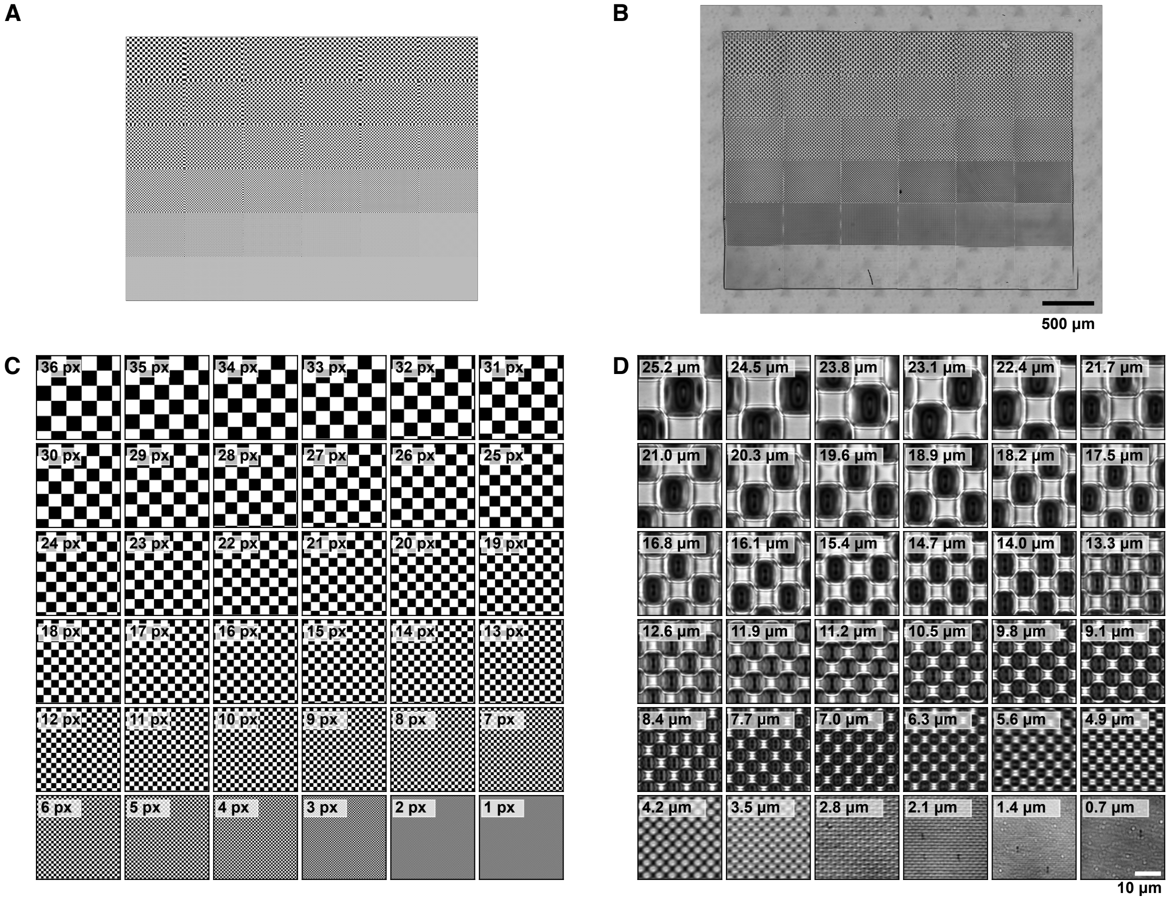

Fig. S3. X/Y resolution test using checkerboard pattern. A 36 computer-designed test masks, containing checkerboard patterns with smaller and smaller squares. Size per square ranges from 36 px in top left corner down to 1 px in bottom right corner. Each of the test masks has a resolution of 800x600 px which corresponds to the resolution of the DMD - one pixel in the mask maps to one pixel of the DMD. **B** Mosaic image of the printed test masks. The masks were printed with a 20x objective on slide spin coated at 3000 RPM. **C** Crop in on the different test patterns of the mask and **D** printed structure. Smallest pattern with clear edges has 4.9 µm pitch. For the checkerboard pattern, the effective resolution we can achieve with the DMD (4.9 µm or 7 px) is smaller than the theoretical resolution (0.7 m or 1 px).

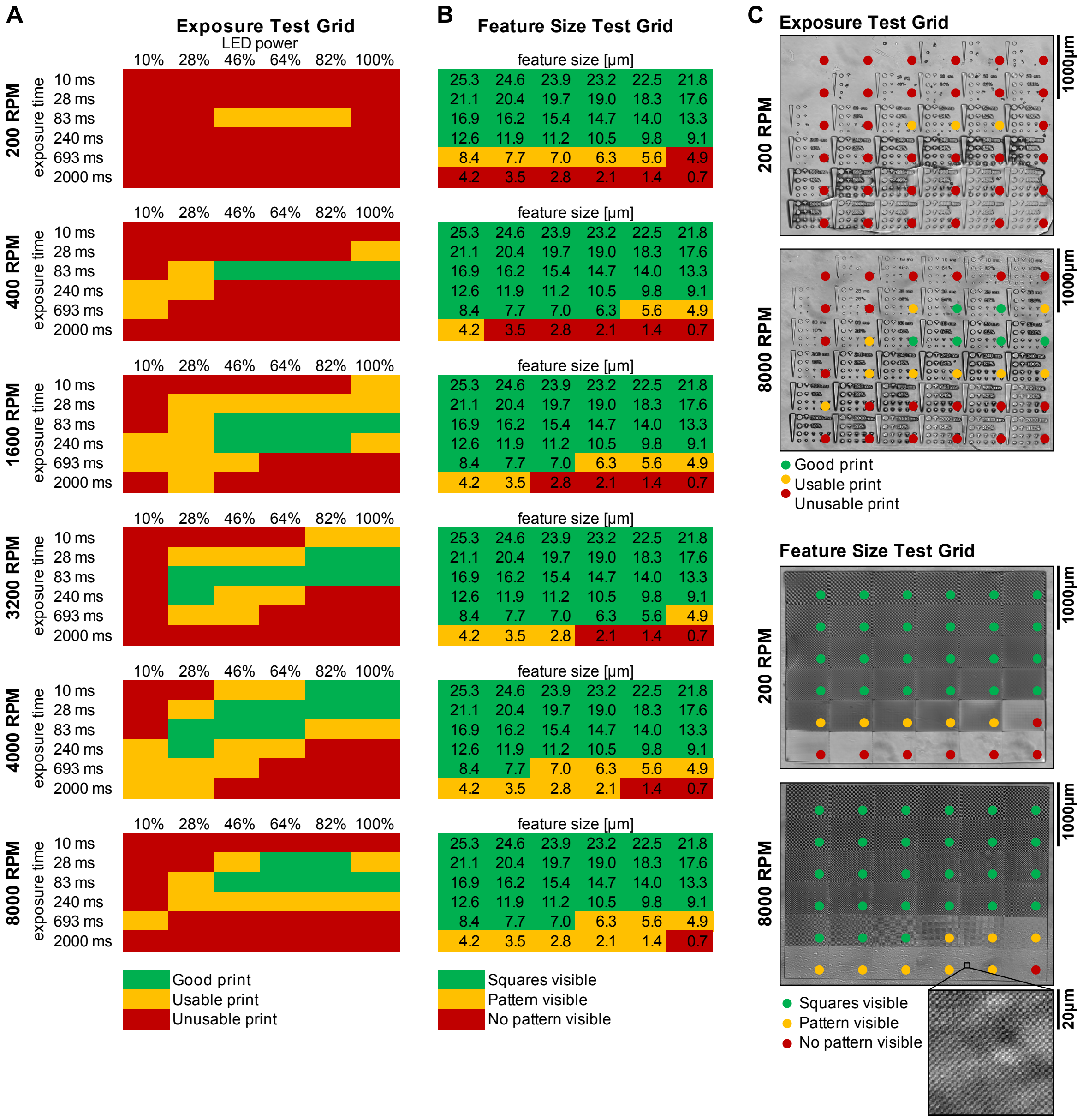

Fig. S4. UV exposure calibration and printing accuracy for different layer heights: **A** UV exposure test grids as shown in supp. Fig. S1 for different spin coating speeds. Colors indicate manual classification of printing quality (green: Good print with no artifacts; orange: Slight artifacts, usable for applications like stamping; red: severe artifacts such as missing features or strong overexposure). With this test pattern, in all samples except 200 RPM good quality prints could be achieved using the same exposure settings (83 ms and 46% / 64% LED power). **B** Checkerboard pattern resolution test as shown in supp. Fig. S3 for different spin coating speeds. Colors indicate manual classification of print quality (green: squares of print mask are clearly replicated; orange: checkerboard structure visible, but appear rounded or deformed; red: features completely merged together, surface appears smooth). **C** Sample test-prints for thickest and thinnest layers (200 and 8000 RPM), shown for both exposure and resolution test grids.

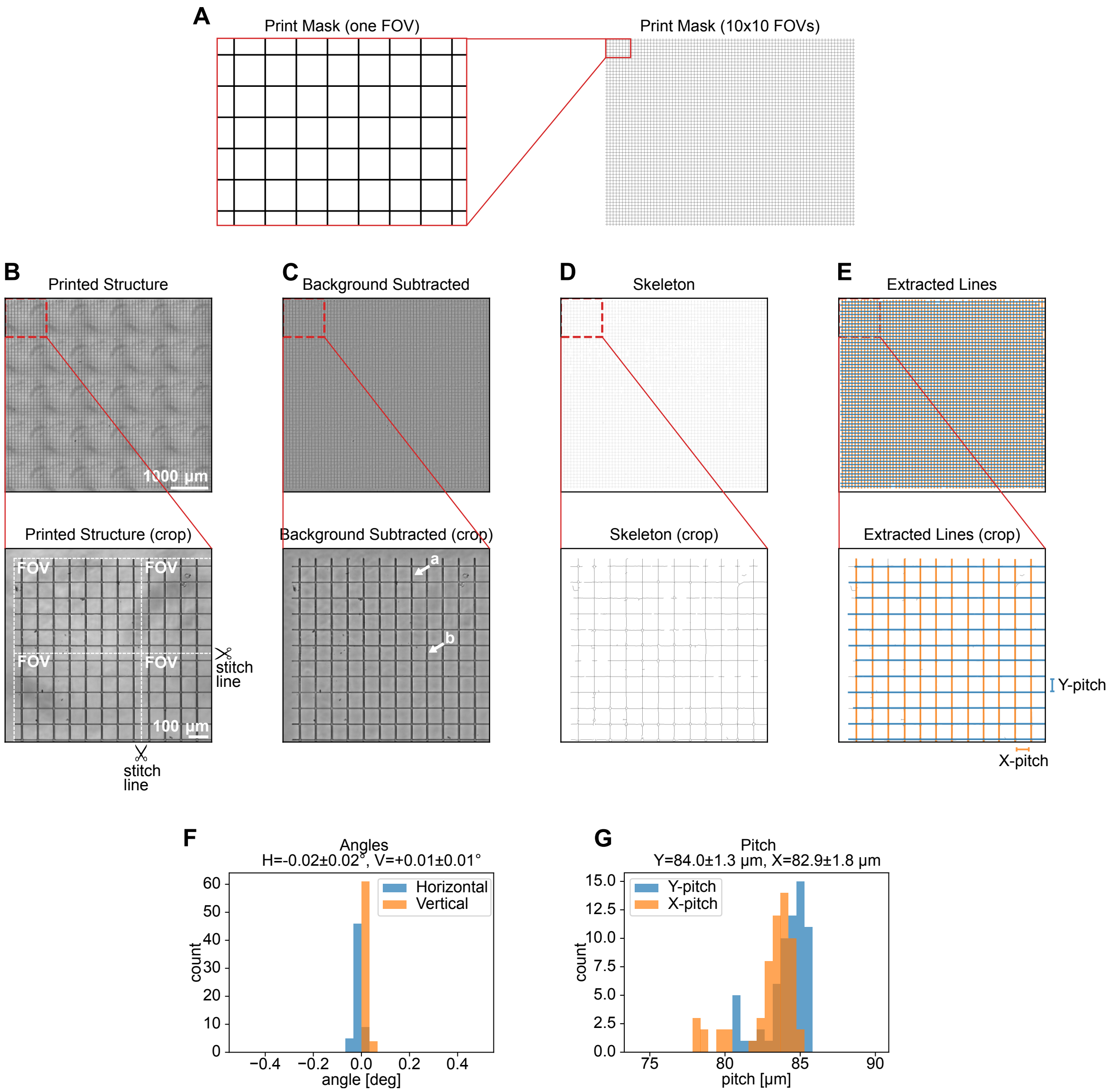

Fig. S5. FOV alignment accuracy over large areas. A Seamlessly tileable grid pattern used to quantify alignment accuracy. A single image and the full pattern repeated 10× in both X and Y directions are shown (final pattern size: 5.6×4.2 mm). **B** Structure printed with a 20× objective and acquired as a mosaic using a 10× objective. Overlay in the crop shows stitching lines between adjacent tiles. **C** High-pass filtered image to remove background artifacts from acquisition. No visible misalignment along vertical stitching lines (arrow a); minor misalignment is visible along horizontal stitching lines (arrow b). **D** Binarized and skeletonized image used for automated line detection. **E** Lines detected using the probabilistic Hough transform (see methods). **F** Histogram of detected line angles: horizontal (H) $-0.02 \pm 0.02^\circ$, $n = 60$; vertical (V) $+0.01 \pm 0.01^\circ$, $n = 65$. **G** Histogram of inter-line distances (pitch): Y-direction 82.9 ± 1.8 μm, $n = 59$; X-direction 84.0 ± 1.3 μm, $n = 64$.

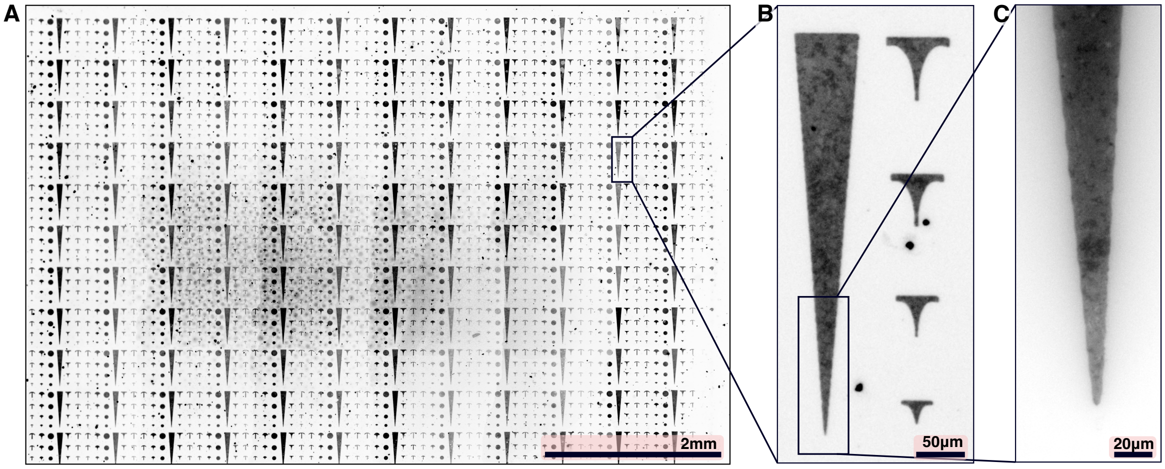

Fig. S6. Micropatterning of large areas: Quality control of the micropatterning workflow using fluorescent laminin. **A**: Mosaic image (10x objective), some over-etching can be observed at the right edge, and some under-etching can be seen in the center of the slide. Different intensities of the patterns are artifacts of the automated image stitching. **B**: Close-up (10x objective). **C**: Close-up of triangle tip (40x objective).

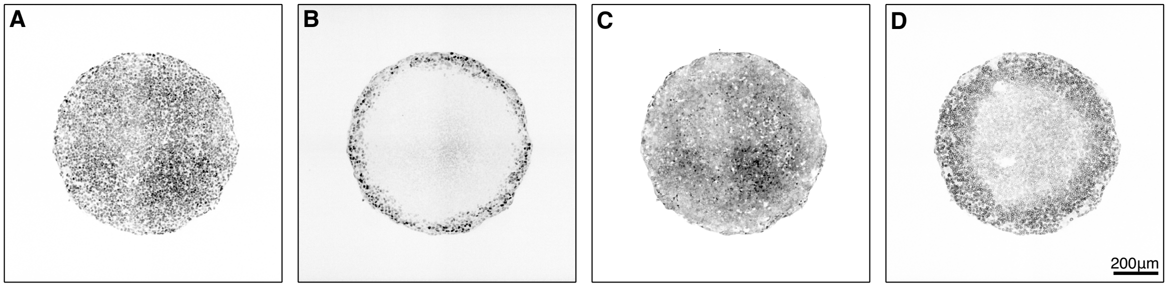

Fig. S7. Micropatterning of 2D gastruloid: 800 μm disk pattern is used to create a circular gastruloid from human embryonic E9 stem cells. This figure shows single channels of composite in Fig. 1B. **A** *H2B::miRFP670* nuclear marker **B** Brachyury immunostain pluripotency marker **C** *ERK-KTR::mClover* **D** *OCT4::POM121::tdTomato*

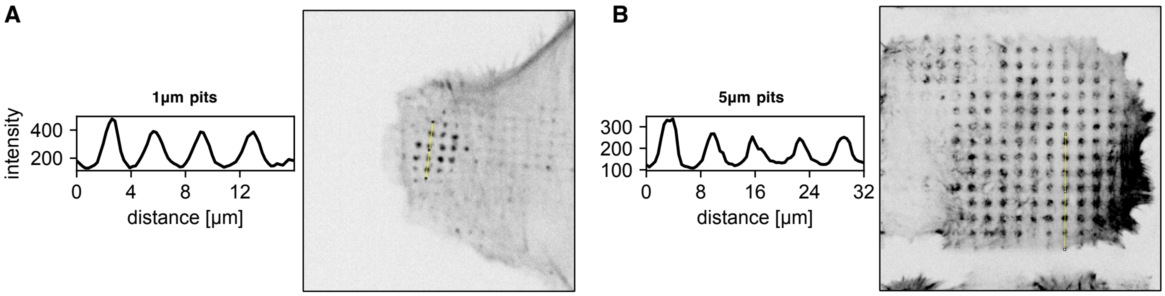

Fig. S8. Actin clusters on microtopology: Lifeact intensity profiles of cells plated on **A** 1 μm sized pits and **B** 1 μm sized pits. Yellow lines indicate location of profile in image.

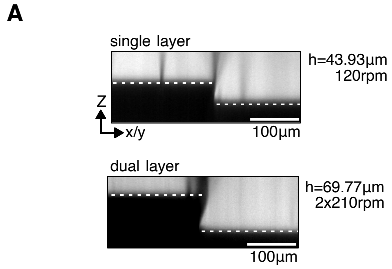

Fig. S9. 2.5D printing: Fluorescence exclusion z-slice of single and dual stacked layers acquire with a confocal microscope.

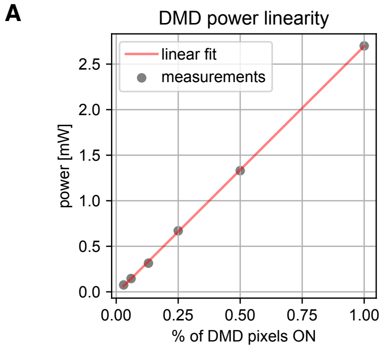

Fig. S10. Measuring DMD output power: Datapoints show light intensity for different percentages of DMD mirrors set to ON state (1/32, 1/16, 1/8, 1/4, 1/2, 1/1). Energy is measured with a light meter held against a 10x objective. A linear fit of the data shows a R^2 value of 1.00 (slope 2.71 mW/100%, intercept -0.02 mW).
